# Fe, Rather Than Soil Organic Matter, as a Controlling Factor of Hg Distribution in Subsurface Forest Soil in an Iron Mining Area

**DOI:** 10.3390/ijerph17010359

**Published:** 2020-01-05

**Authors:** Rui Qu, Guilin Han, Man Liu, Kunhua Yang, Xiaoqiang Li, Jinke Liu

**Affiliations:** Institute of Earth Sciences, China University of Geosciences (Beijing), Beijing 100083, China; qurui@cugb.edu.cn (R.Q.); lman@cugb.edu.cn (M.L.); kunhuayang@cugb.edu.cn (K.Y.); xiaoqli@cugb.edu.cn (X.L.); liujinke@cugb.edu.cn (J.L.)

**Keywords:** mercury, potential ecological risk assessment, iron mining area, spatial distribution, Jiulongjiang River basin, southeast China

## Abstract

To identify whether the iron (Fe) mining area in the Jiulongjiang River basin (JRB) has an influence on the mercury in the forest soil, the spatial distribution patterns of mercury’s behavior on different controlling factors were analyzed, and a potential ecological risk assessment was done. A total of 107 soil samples were collected from two forest soil profiles, one profile near the Fe mining area and the other far from it. The soil near the mining area had a moderate potential ecological risk with high Fe content rich in the upper layer of soil (<70 cm), whereas soil collected far from the mining area had a low potential ecological risk. These results indicated that the rise of iron content in the soil near the mining area was beneficial to the enrichment of mercury, probably causing damage to the forest ecosystem. Both soil organic carbon (SOC) and Fe content have strong positive correlations with THg content, controlling the mercury behavior in the upper layer (<70 cm) and a lower layer (>70 cm) of soil, respectively. The high Fe content in the upper layer of soil will compete for the adsorption of mercury by SOC, leading to the poor correlation between SOC and THg.

## 1. Introduction

Mercury (Hg) has been identified as a global pollutant, with its high neurotoxicity causing adverse effects on the environment and human health [1,2]. The long-distance transport of Hg in the atmosphere, which is emitted by anthropogenic activities and natural sources, causes widespread contamination [3,4]. Soil, generally considered as an essential medium in the ecosystem, has motivated an increasing interest in the sink and the sources of Hg [5,6,7]. Moreover, forest soil, as an active sink of Hg, plays a critical role in global Hg cycling [8]. In a recent study, vegetation was regarded as a large reservoir of Hg in the forest [9]. Ma et al. have corroborated that the forest field had a filtering effect of Hg by comparing Hg deposition fluxes with Hg output stream and runoff. Most Hg ultimately incorporated in the forest floor due to good adsorption of Hg by organic matter and caused a specific ecological risk [10]. With rapid economic growth and iron production in China, Hg accumulation in the surrounding soil has increased [11,12]. Previous reports have studied the iron adsorption of Hg in the soil [13,14]; however, little is understood about the effect of the iron mining area for the Hg in forest soil. Some studies have corroborated that the behavior of Hg has a strong relationship with soil organic carbon (SOC) [15,16], and the forest ecosystem is a significant sink of carbon. As a consequence, the adsorption of mercury by SOC with the influence of the iron mining area in the forest soil profile is worth discussing.

The object of this study is to explore whether the iron content has an influence on the adsorption of mercury by SOC in the forest surface soil near the mining area and determine the potential ecological risk. Moreover, the vertical distribution patterns and the controlling factors of mercury were analyzed to identify the geochemical behaviors in forest soil with the influence of the mining area.

## 2. Materials and Methods

### 2.1. Study Area

**Figure 1:**
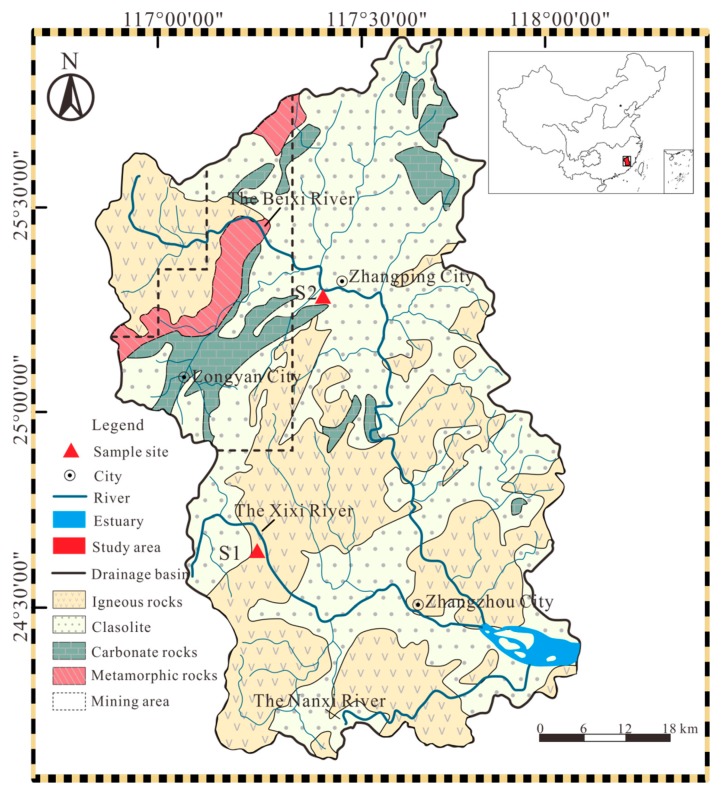
The mining distribution, regional lithology, and location of sampling sites in the Jiulongjiang River.

The Jiulongjiang River is located in the Fujian province, in the southeast of China (24°18′ to 25°88′ N, 116°78′ to 118°03′ E) (Figure 1). The river is a total of 1723 km long, and the size of the drainage basin is 14,700 km^2^ [17]. The Jiulongjiang River consists of three main tributaries (Nanxi River, Xixi River, and Beixi River), which flow into the Xiamen Bay through the regional estuary. The total discharge water is approximately 75% in the catchment with the impact of a seasonal monsoonal climate from April to September [18]. The study area with the subtropical oceanic monsoon climate has an annual temperature from 19.9 °C to 21.1 °C [19]. The terrain in the upper reaches of the river is mountainous, and there is extensive cover of the forest and a few anthropogenic activities in the upper reaches, where the mean annual mean precipitation is about 2000 mm. However, most of the lower reaches have the annual mean precipitation of 1200 mm—related to rural areas, where anthropogenic wastes have been discharged in quantity due to the development of agriculture and industry [20,21]. The increasing precipitation from lower reachers to upper reachers is caused by the NE-trending fault zone and the influence of climate [22].

The Jiulongjiang River basin is an essential tectonic belt due to the interaction between the Eurasian plate and the Pacific plate [23]. With multiple magmatic activities in the Mesozoic, over 60% of the study area consists of intrusive rocks and volcanic rocks in the central and southern parts [24]. Generally, the study area is primarily dominated by carbonate and silicate rocks without obvious evaporite. The carbonate rocks are mainly distributed in the upper reaches of the Beixi River [25], while the silicate rocks are in the lower reaches of the Beixi river, the Xixi River, and the Nanxi River [20]. The area of forest in the Jiulongjiang River basin is 123,000 hm^2^, with a coverage rate of 77.8% [26], and both sampling sites are mixed forests, which primarily consist of evergreen broad-leaf vegetation. Many iron deposits have been found in the study area. The Makeng iron deposit, one of the largest Fe deposits in Fujian province, is a skarn-type deposit near Longyan city [27]. The main types of metal minerals in Makeng iron deposit are magnetite, and galena, sphalerite, and molybdenite [28].

### 2.2. Sampling Collection and Analysis Method

The sampling collection was conducted in the Jiulongjiang River basin (JRB) in January 2018. The locations of two sample sites (S1 and S2) and regional lithology of JRB are shown in Figure 1. The sample sites were both in the forest near the mainstream, and the choice of locations takes into account many factors, such as mining, land use type, and geological conditions. The mining is mainly from the large Fe deposit in Longyan city (Figure 1), where it is close to the sample sites of S2. The land-use type of S1 and S2 is forest, aiming to make a contrast between the north and south of the basin. The sampling information was documented in detail in Table 1, including the record of longitude and latitude coordinates using a global positioning system (GPS). A total of 107 soil samples were collected from soil profiles in both sampling sites, with each soil sample being about 2 kg. The interval of sampling collection was 5 cm in each soil profile. Each soil profile was dug out and the profile was layered according to the color and texture of the soil; then, soil samples were collected from bottom to top, preventing the pollution of soil samples.

After grinding the soil samples to 200 mesh, a machine Retsch MM400 (Retsch GmbH, Haan, Germany) was used to make them homogenized. The total mercury (THg) content was analyzed using an RA-915M mercury analyzer (Lumex Instrument, St.Petersburg, Russia) with a solid module. The accuracy and precision of the mercury analyzer were successfully tested by a previous study [29]. The advantages of using this method to determine the mercury content are speed and its low cost compared to other methods, such as atomic fluorescence spectrometry and inductively coupled plasma optical emission spectrometry (ICP-OES). No digestion of soil samples involved could greatly simplify the mercury test and reduce the mercury losses during pretreatment. During the determination of mercury content, the parallel random samples and standard materials (GBW07402 and GBW07405) were tested every 10 soil samples to ensure the accuracy of the analyzer. The analyzer detection and relative standard deviation (RSD) were 0.10 μg∙kg^−1^ and 4.8%, respectively. As for the test of soil organic carbon, 2 g soil samples were soaked for 24 h with a mixture of 1M KCl and 0.5 M HCl to remove inorganic carbon and inorganic nitrogen [30,31]; then, soil samples were treated with ultrapure water until the pH was neutral. After the pretreatment, SOC was determined by an elemental analyzer (Vario TOC cube; Elementar, Langenselbold, Germany). Besides, the soil was digested using HNO_3_-HF-HClO_4_ and the content of Fe was determined by ICP-MS (Elan DRC-e, Perkin Elmer) [32], and the pH was measured using a pH-meter (INESA Scientific Instrument Co., Ltd., Shanghai, China).

### 2.3. Statistical Analysis

One-way ANOVA was conducted to determine the differences among the THg, SOC, and Fe contents at the upper layer of different sampling sites; statistical significance was at the level of *p* < 0.05. The bivariate correlations with the Pearson correlation coefficient and two-tailed test in line regression analyses were adopted to determine the associations among the THg, SOC, and Fe contents in samples. The data analyses were processed by SPSS 18.0 (SPSS Inc., Chicago, IL, USA) and Microsoft Office 2019 (Microsoft Corporation, Redmond, Seattle, WA, USA). The figures were performed by Origin 9.0 (OriginLab Corporation, Northampton, MA, USA) and SigmaPlot 12.5 (Systat Software GmbH, Erkrath, Germany) software packages.

### 2.4. Potential Ecological Risk Assessment

The potential ecological risk index ($E_{r}^{i}$) has been an effective tool to assess the ecological risks of heavy metals. The risks caused by mercury contamination in this study area was assessed with the following equation:

(1) $$E_{r}^{i}=T_{r}^{i}\times \frac{C^{i}}{C_{n}^{i}},$$

where $C_{n}^{i}$ represents the background value of metal n in the study area; $T_{r}^{i}$ (usually 40 for mercury) represents toxic the response factor; and *C^i^* represents the content of samples for metal *i*. Generally, the potential ecological risk index values were classified into five categories: (i) low potential ecological risk ($E_{r}^{i}$ < 40); (ii) moderate potential ecological risk (40 ≤ $E_{r}^{i}$ < 80); (iii) considerable potential ecological risk (80 ≤ $E_{r}^{i}$ < 160); (iv) high potential ecological risk (160 ≤ $E_{r}^{i}$ < 320); (v) very high ecological risk ($E_{r}^{i}$ ≥ 320) [33].

## 3. Results and Discussion

### 3.1. Overview of THg in Soil Profiles

An overview of THg and other contents in both sampling sites is provided in Table 2, and the orginal data is given in Appendix A Table A1. The maximum mercury content in the studied soils is higher than the background mercury content, 81 μg∙kg^−1^ [34], especially in the S2 profile. Studies have compared SOC content and pH values in both soil profiles and found that they are essentially identical. Data on both profiles fluctuate largely with respect to Fe content due to the influence of anthropogenic activities and presentation of iron rust.

**Figure 2:**
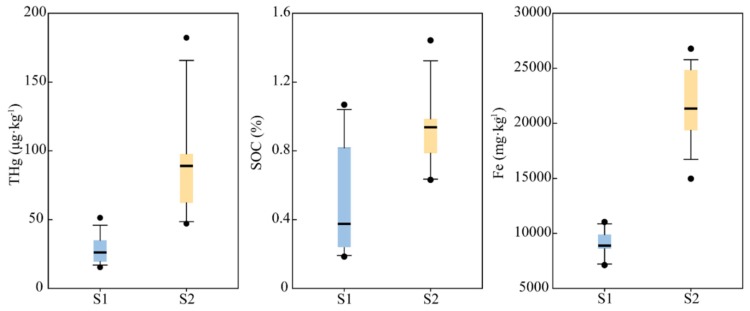
The contents of THg, SOC, and Fe in each profile (0–70 cm) (o represents an outlier).

For the upper layer of soil profiles (<70 cm), there are significant differences in the two sampling sites (Figure 2). In general, the contents of THg, SOC, and Fe in the S2 profile are higher than that in the S1 profile, especially for the THg and Fe contents. The amount of Fe concentrates in the upper layer of the S2 profile in comparison to the S1 profile, attributed to the location of the S2 site close to the mining area. A probable explanation is that this enrichment of Fe content in the mining area is a result of high mercury content in the S2 profile with respect to the S1 profile.

### 3.2. THg Distribution Pattern and Controlling Factors in Soil Profiles

**Figure 3:**
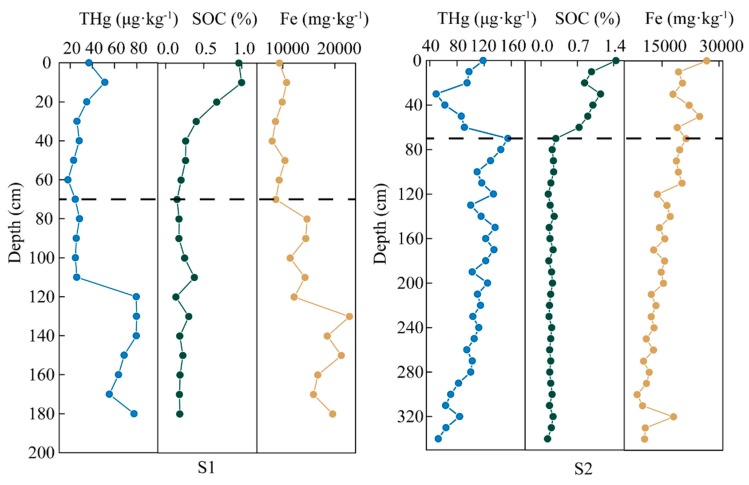
Vertical distribution patterns in forest soil profiles, including THg, SOC, and Fe contents.

The vertical distribution patterns of total mercury, divided into a upper layer (<70 cm) and a lower layer (>70 cm) of soil in Figure 3, show a significant discrepancy in different forest soil profiles in the study area. Generally, the THg content of both profiles is a little higher in the surface soil (<2 cm), perhaps attributable to the cover of the plant to avoid the solar radiation [35]. The THg content is more homogenous in the S1 profile than that in the S2 profile, even though there is a significant rise of THg content at a depth of 120 cm in the S1 profile. At the depth from 70 to 110 cm, the THg content in the S1 profile slightly increases. Compared to the S1 profile, the THg content in the S2 profile shows complicated variations in the upper layer, and it decreases with the depth in the lower layer of soil. However, the soil organic carbon shows a similar variation as the content decreases with depth in the upper layer, and it becomes homogenous in the lower layer, which is an agreement with a previous study [36]. The SOC content in the S2 profile stops reducing at the end of upper layer of soil and becomes steady; thus, the division of the soil profile is conducted according to the influence of the SOC content. The vertical distribution patterns of SOC in the forest soil profiles are in agreement with the previous reports [37,38]. As for the vertical distribution of iron (Fe) content, the Fe content is higher in the S2 profile than that in the S1 profile, and decreases with the depth, while the Fe content is concentrated in a lower layer of the S1 soil profile. There is a significant increase at the end of the upper layer in the S1 soil profile with the observation of the occurrence of iron rust.

**Figure 4:**
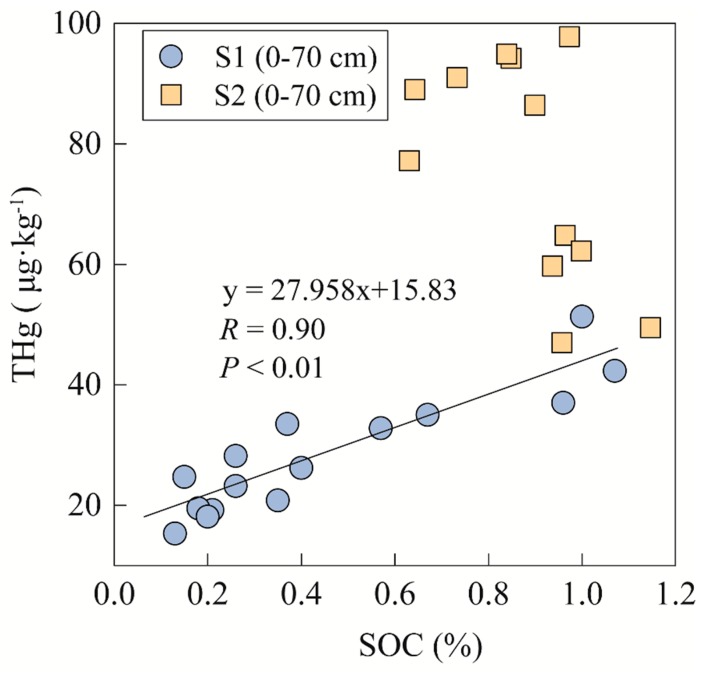
Line regression relationships between the SOC content and THg content in the upper layers (<70 cm) of both forest soil profiles.

What is striking in this Figure 3 is the different THg distributions of the S1 profile and S2 profile, which have motivated interest in the controlling factors for these patterns. Several factors are known to play a role in determining mercury distribution, such as SOC content and Fe content. Soil organic carbon can enhance the adsorption of metals for soil to make the soil mercury concentrated [39,40]. The ionic mercury can be combined strongly by organic molecules, such as humic acids and fulvic acids in the presence of soil organic matter [41]. Hg tends to be complexed with S-rich varieties in organic matter, which have a high affinity for mercury, resulting in Hg accumulation in the soil [42,43]. In the upper soil, the SOC content is usually taken into consideration for the controlling factor of mercury in soil with high correlations between THg content and SOC content [44,45,46]. However, as shown in Figure 4, soil organic carbon content shows a significant correlation (*R* = 0.90, *p* < 0.01) with THg content in the upper soil of the S1 profile, and that in the S2 profile is in contrast. This discrepancy could be attributed to the decomposed of light fraction of SOC. The soil microorganisms under forest soil easily decompose the light fraction of SOC [47]; thus, the decomposed light fraction under forest cover probably has an influence on the Hg/SOC ratio, resulting in a low correlation between THg content and SOC content [46]. Moreover, the THg content in the S2 profile, where there is higher SOC content compared to that in the S1 profile, shows no significant correlation with SOC content, probably implying that there is another competitively controlling factor for mercury. It is likely the high Fe content caused by the mining area affects the adsorption for mercury from SOC. In the upper soil, the Fe content is almost double, in the S2 profile, that in the S1 profile, which may have caused the discrepancy.

**Figure 5:**
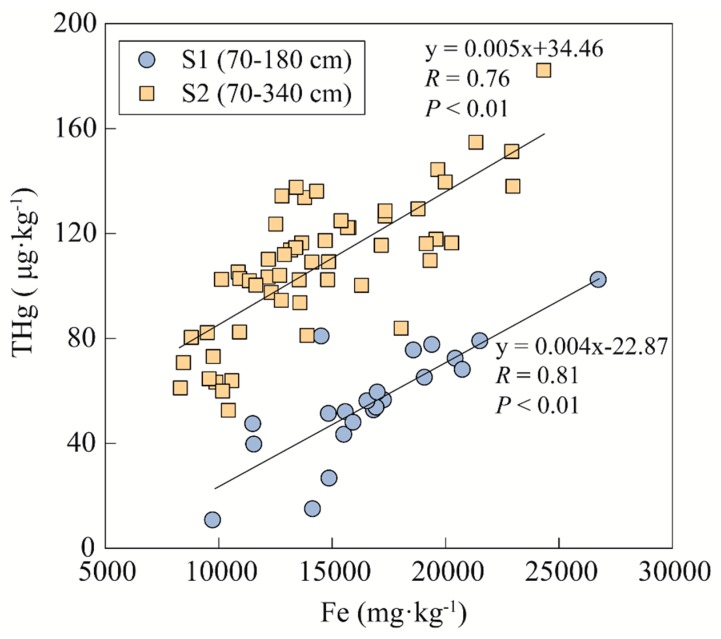
Line regression relationships between the Fe content and THg content in the lower layers (>70 cm) of both forest profiles.

Some studies have been reported wherein the vertical distribution pattern of THg content decreases with depth [48,49]. However, the vertical distribution patterns of mercury in our study area shows enrichment in the lower layer of the soil profile to some extent. The iron rust presentation in the lower layer of the S1 profile results in the high Fe content, while the Fe is concentrated in the upper layer of the S2 profile due to the influence of the mining area. The high similarity of the distribution pattern of Fe and Hg in S2 (>20 cm) indicates that the main interaction of Hg in the soil is probably with Fe oxides. Fe oxides are good adsorbents for heavy metals, exhibiting specific adsorption of mercury through ion exchange [50]. Besides, the presence of Fe is an important abiotic factor for Hg oxidation from Hg^0^ to Hg^2+^ [51], which can lead to Hg immobilization. As a high Fe content is beneficial for the adsorption for mercury in soil [52], the increase of Fe content has an influence on mercury’s distribution behavior. The correlations between Fe content and THg content have been analyzed for the lower layers of both profiles, as shown in Figure 5. The strong correlations indicate that the Fe content has contributed to a rise of mercury content in the soil profiles. These results do not rule out the other factors in the vertical distribution of mercury. However, after the SOC content decreases, the Fe becomes the dominant controlling factor on the vertical distribution of mercury, affecting the enrichment of THg in both forest profiles.

### 3.3. Potential Ecological Risk of Hg in Soil Profiles

**Figure 6:**
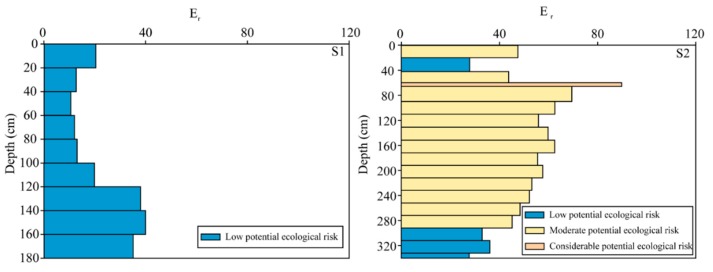
The ecological assessment in S1 and S2 forest soil profiles.

In order to conduct the aim of risk evaluation in the forest soil profiles, the ecological assessment of mercury was performed, as shown in Figure 6. It is apparent that all the soil samples in S1 profiles present low potential ecological risk with their slightly low mercury contents. However, over two-thirds of soil samples present moderate potential ecological risk; some soil samples even present a considerable potential ecological risk. These results probably indicate that the extremely mercury contamination occurs in the north of JRB due to the mining, whereas the contamination is comparatively slight in the south of JRB.

## 4. Conclusions

This study has shown that the risk evaluation of mercury, using geochemical characteristics of mercury distribution in relation to different controlling factors in forest soil profiles in the Jiulongjiang River basin, southeast China. The mercury content tents to be enriched in the lower layer (>70 cm) due to the rise of Fe content in soil profiles. In the upper layer (<70 cm) of the soil profile, the dominant controlling factor of mercury content is SOC content, with competition from Fe. However, in the lower layer of the soil profile, the main controlling factor is Fe exclusively, as the SOC content decreases. The high Fe content near the mining area in the upper layer of soil will compete for the adsorption of mercury with SOC, leading to the poor correlation between SOC and THg. In the north of the Jiulongjiang River basin, there is a moderate potential ecological risk of mercury and even a little bit of a considerable potential ecological risk from mercury in the forest soil profile, due to the mining area of Fe. The high Fe content has probably contributed to the enrichment of mercury, causing potential contamination. However, with relatively low mercury in the south of the Jiulongjiang River basin, there is a low potential ecological risk from mercury. According to our results, the contamination caused by the mining area has a strong influence on the near ecological environment, so it should be focused on and monitored. Further study about the light fraction of SOC and the associations between species of mercury and Fe content will be conducted to verify the adsorption of Hg by Fe and explore the mechanism.

## Figures and Tables

**Table 1:** The information regarding the sampling sites.

Sampling Site	Location	Main Plant	Depth (cm)	Visible Characteristic of Soil
S1	117°14′5″ E, 24°39′6″ N	Myrtle (*Rhodomyrtus tomentosa*), masson pine (*Pinus massoniana Lamb*)	0–40 40–70 70–180	Black, humus layer Gray, silty loam Red, silty clay loam, iron rust
S2	117°25′28″ E, 25°16′21″ N	Pine (*Pinus tabuliformis Carr*)	0–65 65–120 120–235 235–340	Gray–red, humus layer red, loam Crimson, loam light red, loam

**Table 2:** Statistics of the data for Hg (μg∙kg^−1^), soil organic carbon (SOC—%), Fe (mg∙kg^−1^), and pH (in unit).

		*n*	Min	Max	Mean	SD
S1	Hg	37	15.30	107.10	44.92	25.71
	SOC	37	0.12	1.07	0.31	0.24
	Fe	37	7115.00	27,550.00	13,653.73	5041.15
	pH	37	4.34	5.11	4.67	0.18
S2	Hg	69	47.00	182.20	102.46	27.84
	SOC	69	0.10	1.44	0.33	0.31
	Fe	69	8312.00	26,780.00	15,682.03	4741.00
	pH	69	4.08	4.56	4.33	0.13

## Appendix A

**Table A1:** Data for Hg, SOM, and Fe contents in each soil layer.

Sampling Site	Depth (cm)	Hg (μg∙kg^−1^)	SOM (%)	Fe (mg∙kg^−1^)
S1	0	37.0	1.0	9368
	5	42.3	1.1	8855
	10	51.3	1.0	10,750
	15	32.8	0.6	8797
	20	35.0	0.7	9893
	25	20.8	0.4	7282
	30	26.2	0.4	8612
	35	33.5	0.4	11,030
	40	28.2	0.3	7986
	45	19.2	0.2	8871
	50	23.2	0.3	10,420
	55	19.5	0.2	7115
	60	18.1	0.2	9315
	65	15.3	0.1	8990
	70	24.7	0.1	8684
	75	26.0	0.2	13,090
	80	28.5	0.2	14,660
	85	25.6	0.1	11,560
	90	25.6	0.2	14,430
	95	25.8	0.2	13,800
	100	24.8	0.2	11,420
	105	23.2	0.1	12,910
	110	26.0	0.4	14,270
	115	35.6	0.2	15,040
	120	79.7	0.1	12,200
	125	60.0	0.3	18,860
	130	79.8	0.3	22,830
	135	88.6	0.2	22,460
	140	79.7	0.2	18,560
	145	84.9	0.2	15,490
	150	68.7	0.2	21,270
	155	107.1	0.2	27,550
	160	63.6	0.2	16,740
	165	84.4	0.2	19,750
	170	55.3	0.2	15,910
	175	64.3	0.2	16,810
	180	77.6	0.2	19,610
S2	0	118.5	1.4	26,780
	5	94.2	0.8	25,130
	10	97.8	1.0	19,370
	15	77.2	0.6	22,200
	20	94.9	0.8	20,390
	25	64.8	1.0	24,870
	30	49.5	1.1	17,900
	35	47.0	1.0	14,960
	40	62.2	1.0	22,160
	45	59.7	0.9	20,090
	50	86.4	0.9	24,900
	55	89.0	0.6	20,100
	60	91.0	0.7	19,020
	65	182.2	0.4	24,330
	70	154.8	0.3	21,340
	75	138.0	0.2	22,970
	80	144.4	0.2	19,660
	85	126.7	0.2	17,330
	90	129.4	0.2	18,790
	95	128.6	0.2	17,340
	100	109.7	0.2	19,320
	105	151.3	0.2	22,920
	110	116.4	0.2	20,270
	115	139.6	0.2	19,990
	120	133.7	0.1	13,790
	125	80.4	0.2	8795
	130	100.2	0.2	16,300
	135	117.8	0.2	19,580
	140	115.5	0.3	17,170
	145	116.1	0.2	19,150
	150	136.1	0.2	14,320
	155	113.7	0.1	13,170
	160	122.2	0.2	15,740
	165	137.6	0.2	13,420
	170	134.3	0.2	12,790
	175	117.3	0.2	14,700
	180	122.2	0.1	15,680
	185	109.2	0.2	14,850
	190	102.4	0.2	14,800
	195	109.1	0.2	14,110
	200	124.9	0.2	15,390
	205	123.6	0.3	12,520
	210	110.2	0.2	12,190
	215	116.4	0.3	13,660
	220	114.6	0.2	13,400
	225	97.5	0.2	12,320
	230	103.4	0.2	12,180
	235	104.0	0.2	12,690
	240	112.0	0.2	12,900
	245	102.3	0.1	13,550
	250	105.3	0.2	10,860
	255	102.8	0.1	10,930
	260	94.5	0.2	12,760
	265	93.6	0.1	13,580
	270	102.5	0.2	10,130
	275	102.0	0.3	11,350
	280	100.3	0.2	11,640
	285	82.2	0.2	9498
	290	82.4	0.2	10,920
	295	73.1	0.2	9759
	300	70.8	0.2	8448
	305	61.1	0.1	8312
	310	63.3	0.2	9878
	315	81.1	0.2	13,900
	320	83.9	0.2	18,040
	325	64.6	0.2	9580
	330	63.9	0.2	10,580
	335	59.9	0.2	10,170
	340	52.6	0.1	10,430
